# Multidetector CT with 3-dimensional volume rendering in the evaluation of the spine in patients with Neurofibromatosis type 1: a retrospective review in 73 patients

**DOI:** 10.1186/1748-7161-9-15

**Published:** 2014-09-23

**Authors:** James Matthew Debnam, Yasser MM Mahfouz, Leena Ketonen, John M Slopis, Ian E McCutcheon, Nandita Guha-Thakurta

**Affiliations:** 1 Department of Radiology, Section of Neuroradiology, The University of Texas M.D. Anderson Cancer Center, 1515 Holcombe Blvd., Unit 370, Houston, TX 77030, USA; 2 Department of Radiology, National Cancer Institute, Cairo, Egypt; 3 Department of Neuro-Oncology, The University of Texas M.D. Anderson Cancer Center, Houston TX 77030, USA; 4 Department of Neurosurgery, The University of Texas M.D. Anderson Cancer Center, Houston, TX 77030, USA

**Keywords:** Abnormalities, Computed tomography, Computer-assisted three dimensional imaging, Neurofibromatosis 1, Spine

## Abstract

**Introduction:**

Neurofibromatosis type 1 (NF-1) may involve the spine as various abnormalities including bony dysplasia, scoliosis, and nerve sheath tumors. Surgery may be performed for stabilization of the spine. We have seen an increase in requests for multidetector CT (MDCT) imaging with the (three-dimensional) 3D-volume rendered (VR) images in patients evaluated at our institution. We, therefore, investigated how MDCT could be best utilized in this patient population.

**Methods:**

Seventy-three patients with NF-1 were identified in whom MDCT imaging was performed for diagnostic, pre-operative, or post-operative evaluation of spinal abnormalities. True axial source images and two dimensional (2D) orthogonal reconstructed MDCT images, as well as the VR images, were compared with plain radiographs and MRI. In addition, the MDCT study was compared to the VR images. These studies were reviewed to compare assessment of A) bony abnormalities such as remodeling from dural ectasia, dysplasia, and fusion, B) abnormal spinal curvature, C) nerve sheath tumors, and D) surgical instrumentation.

**Results:**

When compared to plain radiographs, the MDCT and VR images were rated as helpful for evaluating the abnormalities of the spine in 19 of 24 patients for a total of 30 findings. This included the following categories A) (n = 6), B) (n = 5), C) (n = 7), and D) (n = 12). Compared to MR, the MDCT and VR study was helpful in evaluating the findings of NF-1 in 24 of 36 patients for a total of 40 findings. This included the following categories A) (n = 12), B) (n = 10), C) (n = 3), and D) (n = 15). When the VR images were compared to the orthogonal MDCT, the VR images was rated as helpful in 41 of 73 patients for a total of 60 findings, including the following categories: A) (n = 11), B) (n = 24), C) (n = 0), and D) (n = 25).

**Conclusion:**

MDCT has distinct advantages over plain radiographs and MR imaging, and the VR images over MDCT in the evaluation of the spine in patients with NF-1, especially for the assessment of bony abnormalities, abnormal spinal curvature, and spinal instrumentation.

## Background

Neurofibromatosis type 1 (NF-1) is one of the most common genetic disorders, affecting between 1 in 3000 to 3500 individuals [1,2]. NF-1 is caused by defect in the gene responsible for the production of the protein neurofibromin, a tumor suppressor gene linked to the long arm of chromosome 17 [3]. Inheritance is autosomal dominant, although about 50% of cases develop sporadically as new mutations [2]. The clinical diagnosis is made according to the diagnostic criteria established by the National Institute of Health Consensus Development Conference in 1987 [4].

When a neurological deficit is present in the spine in a patient with a neurofibromatosis, the cause should accurately be defined to prevent serious complications that may be related to spinal deformity or structural instability [5-7]. Evaluation of patients with NF-1 has routinely been performed with plain film radiographs and MRI [5,8-12]. However, computed tomography (CT) has recently been described for the assessment of an abnormal spinal curvature [13-20].

Advances in multidetector CT (MDCT) technology allow computer manipulation of the axial CT source data and generation of orthogonal two-dimensional (2D) images and three-dimensional (3D) volume rendered (VR) images. These VR images demonstrate the surface of the vertebral body, and a translucent display allows visualization of surgical instrumentation through the vertebral bodies. The VR images, which do not require additional radiation, can be rotated and viewed from 360 degrees, have led to an increased demand for MDCT imaging of the spine at our institution, a major cancer center. In fact, MDCT is now the standard of care for assessing the spine in patients with trauma in many emergency departments [21,22].

To the best of our knowledge, no reports exist in the literature on the use of MDCT with the VR series, including the translucent display, for evaluation of the spine in patients with NF-1. As additional time for processing and interpretation of the 3D VR images are required, we sought to determine which characteristics would be best assessed by MDCT with the 3-D VR images. To do this, we compared MDCT with the VR series to plain radiographs and MRI, as well as MDCT to the VR images of the spine in patients with NF-1. The purpose was not to test which of the aforementioned modalities best evaluates certain abnormalities compared to MDCT, rather to determine where MDCT and the VR images may provide the most information, thus benefiting this patient population.

## Methods

The Institutional Review Board at The University of Texas MD Anderson Cancer Center approved this study and waived the requirement for informed consent. Data acquisition was performed in compliance with all applicable Health Insurance Portability and Accountability Act regulations. MDCT examinations of the spine, performed at our institution between 2003 and 2013, were retrospectively reviewed. Inclusion criteria for this study included patients with NF-1 for whom MDCT was requested.

Plain radiographs, MR examinations, and MDCT examinations, including with sagittal and coronal reformatted, and VR images were retrospectively reviewed by 3 neuroradiologists (JMD, LK and NGT) by consensus. The reviewers evaluated abnormalities on the studies to determine if MDCT with the VR images demonstrated findings better than on plain films and MR in the following categories: A) bony abnormalities such as remodeling from dural ectasia, dysplasia, and fusion, B) abnormal spinal curvature, C) nerve sheath tumors, and D) instrumentation. In addition, MDCT was compared to the VR images in the aforementioned 4 categories.

The MR examinations included the following parameters: sagittal T1-weighted (TR, 400–650 ms; TE, 9–19 ms) and fast spin echo (FSE) T2- weighted (TR, 3000–6100; TE, 90–110 ms), axial T1 pre-gadolinium (TR, 350–850 ms; TE 9–14 ms), axial T1 post gadolinium (TR 400-750ms; TE 9-19ms) and sagittal T1 post gadolinium (TR 400–800; TE 9-18ms). Axial imaging was acquired at 4–5 mm section thickness with section gap of 1 mm and sagittal imaging was acquired at a slice thickness of 5–8 mm with section gap of 1–2 mm and. Intravenous gadolinium (Omniscan, GE Medical Systems) was administrated in all cases.

The MDCT examination was performed on a multidetector CT scanner (GE Medical Systems), with the following parameters: 140 kV, 220 to 250 mA, and a 1.25-mm slice thickness. Post-processing was performed by a trained technologist on an Advantage AW4.2 workstation (GE Medical Systems) using Volume View software (GE Medical Systems). The MDCT scans were acquired without, or without and with intravenous contrast (Optiray, Mallinckrodt Inc., St. Louis, MO). Bone algorithm and soft tissue windows were available and reviewed in all cases. The post-processing provided imaging in the sagittal and coronal planes in all patients. The 3-D VR images with translucent display was available for patients with surgical instrumentation.

## Results

Seventy-three consecutive patients (52 female, 21 male), age 7–81 years (median age 38 years ± 15.3 years) with NF-1 and MDCT imaging of the spine were identified and included in this study. The patient demographics are included in Table 1.

**Table 1:** Patient demographics and associated abnormality

**Patient #**	**Age**	**Sex**	**Abnormality**
1	19	F	Neurofibrosarcoma
2	14	F	Neurofibrosarcoma
3	70	F	nst
4	30	M	nst, rib abnormalities
5	42	M	None
6	44	F	nst, instrumentation
7	44	F	nst, instrumentation
8	37	F	nst
9	32	F	nst
10	24	F	Scoliosis
11	55	F	Scoliosis
12	43	M	Scoliosis, instrumentation
13	12	M	Scoliosis, nst
14	63	F	Scoliosis, nst
15	18	F	Scoliosis, nst
16	38	F	Bony fusion, dural ectasia, nst, scoliosis, instrumentation
17	36	F	Bony fusion, dural ectasia, scoliosis, instrumentation
18	42	F	Dural ectasia, scoliosis
19	20	F	Dural ectasia, wedge deformity, scoliosis, nst
20	20	F	Scoliosis, instrumentation
21	20	F	Scoliosis, instrumentation
22	16	M	nst
23	29	F	Bone dysplasia, scoliosis
24	54	F	Bone dysplasia, scoliosis
25	44	F	Scoliosis, instrumentation
26	40	F	Bone fusion, scoliosis
27	55	F	None
28	39	F	Instrumentation
29	56	F	Scoliosis, instrumentation
30	63	M	Instrumentation
31	30	F	Bone fusion, scoliosis
32	38	F	Post laminectomy
33	27	F	Post laminectomy
34	81	F	Scoliosis
35	20	F	Bone fusion, scoliosis, instrumentation
36	29	F	Scoliosis, instrumentation
37	24	F	Scoliosis
38	28	M	Post laminectomy
39	56	F	Instrumentation
40	19	F	nst
41	27	M	Instrumentation
42	53	F	None
43	47	M	Post laminectomy
44	28	M	nst
45	16	F	Instrumentation
46	54	F	Bone dysplasia, scoliosis
47	28	F	Instrumentation
48	38	M	Soft tissue sarcoma
49	23	M	Instrumentation
50	40	F	nst
51	49	F	Instrumentation
52	31	F	None
53	20	M	nst
54	26	M	Scoliosis
55	48	M	nst
56	39	F	Scoliosis, instrumentation
57	49	F	Instrumentation
58	45	M	None
59	45	F	None
60	53	M	Instrumentation
61	39	M	Scoliosis
62	51	F	Scoliosis
63	28	F	Instrumentation
64	27	F	Scoliosis, instrumentation
65	22	F	Instrumentation
66	43	F	Instrumentation
67	49	F	Instrumentation
68	47	M	Scoliosis
69	7	M	Instrumentation
70	13	F	Instrumentation
71	38	M	nst
72	45	F	Scoliosis
73	9	F	Scoliosis

nst: nerve sheath tumor.

Two-view plain radiographs of the spine were also obtained in 24 patients between 0 and 132 days (median 0 days ± 26.3 days) to when the MDCT study was performed. When compared to plain radiographs, the MDCT and VR images were rated as better in the evaluation of findings in 19 of 24 patients for a total of 30 findings. This included the following categories A) bony abnormalities (n = 6), B) abnormal spinal curvature (n = 5), C) nerve sheath tumors (n = 7), and D) instrumentation (n = 12). More specific information is available in the Table 2.

**Table 2:** MDCT/VR added to plain radiographs

**Patient #**	**CT/VR additional findings**
1	Vertebral body and transverse process destruction
2	Sacral destruction, nst
3	nst
7	nst, instrumentation
12	Scoliosis, instrumentation
13	nst
16	Dural ectasia, bony fusion, scoliosis,
	nst, instrumentation
17	Dural ectasia, bony fusion,
	Scoliosis, instrumentation
22	nst
24	Bone dysplasia, scoliosis
25	Scoliosis, instrumentation
28	Instrumentation
30	Instrumentation
41	Instrumentation
47	Instrumentation
48	Soft tissue sarcoma
49	Instrumentation
60	Instrumentation
63	Instrumentation

nst: nerve sheath tumor.

MR examinations were performed in 36 patients between 1 and 183 days (median 0 days ± 29.3 days) in relation to when the MDCT examinations were acquired. The MDCT and VR study was better than MR in evaluating the findings of NF-1 in 24 of 36 patients for a total of 40 findings, including the following categories A) bony abnormalities (n = 12), B) abnormal spinal curvature (n = 10), C) nerve sheath tumors (n = 3), and D) instrumentation (n = 15). More specific information is available in the Table 3.

**Table 3:** MDCT/VR added to MRI

**Patient #**	**CT/VR additional findings**
1	Vertebral body and transverse
	Process destruction
4	Ribbon ribs
6	nst, instrumentation
11	Scoliosis
12	Scoliosis, instrumentation
13	Transverse process destruction, nst
16	Bony fusion, dural ectasia
	Scoliosis, instrumentation
17	Bony fusion, dural ectasia
	Scoliosis, instrumentation
18	Dural ectasia, scoliosis
19	Dural ectasia, wedge deformity, scoliosis
22	nst
24	Bone dysplasia, scoliosis
25	Scoliosis, instrumentation
28	Instrumentation
45	Instrumentation
46	Bone dysplasia, scoliosis
47	Instrumentation
49	Instrumentation
51	Instrumentation
57	Instrumentation
60	Instrumentation
63	Instrumentation
64	Scoliosis, instrumentation
66	Instrumentation

nst: nerve sheath tumor.

When the VR images were compared to the orthogonal MDCT in all 73 patients, it was rated as helpful in 41 of 73 patients for a total of 60 findings, including the following categories: A: bony abnormalities (n = 11), B: abnormal spinal curvature (n = 24), C: nerve sheath tumors (n = 0), and D) instrumentation (n = 25). More specific information is available in the accompanying Table 4.

**Table 4:** Volume rendered series added to MDCT

**Patient #**	**VR additional information**
1	Transverse process destruction
10	Scoliosis
11	Scoliosis
12	Scoliosis, instrumentation
13	Transverse process destruction
	Scoliosis
15	Scoliosis
16	Bony fusion, dural ectasia
	Scoliosis, instrumentation
17	Bony fusion, dural ectasia
	Scoliosis, instrumentation
18	Scoliosis
19	Wedge deformity, scoliosis
24	Scoliosis
25	Scoliosis, instrumentation
26	Bony fusion, scoliosis
28	Instrumentation
29	Scoliosis, instrumentation
30	Instrumentation
31	Bone fusion, scoliosis
33	Post laminectomy
35	Bone fusion, scoliosis
	Instrumentation
36	Scoliosis, instrumentation
41	Instrumentation
45	Instrumentation
46	Bone dysplasia, scoliosis
47	Instrumentation
49	Instrumentation
51	Instrumentation
56	Scoliosis, instrumentation
57	Instrumentation
60	Instrumentation
61	Scoliosis
62	Scoliosis
63	Instrumentation
64	Scoliosis, instrumentation
65	Instrumentation
66	Instrumentation
67	Instrumentation
68	Scoliosis
69	Instrumentation
70	Instrumentation
72	Scoliosis
73	Scoliosis

## Discussion

Our results demonstrated that more information can be obtained when MDCT is performed in addition to plain radiographs and MRI in patients with NF-1. Furthermore, our results show that the VR images provide additional information to that of MDCT alone. We found MDCT and the VR images to be particularly beneficial, in the evaluation of bony abnormalities such as remodeling from dural ectasia, bone dysplasia and fusion, abnormal spinal curvature, and spinal instrumentation, but less so for nerve sheath tumors.

**Figure 1:**
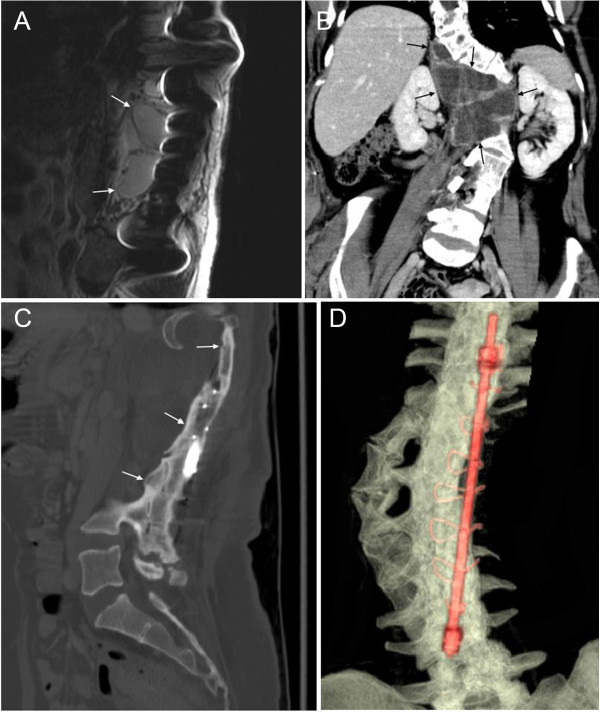
**A 20 year-old women (Patient #18) with large neurofibromas and vertebral body dysplasia. A)** Sagittal T1 postcontrast MRI demonstrates neurofibromas (arrows). **B)** Axial T1 postcontrast MR shows the neurofibromas (large arrows) and suggests that a rotatory scoliosis exists. **C, D)** 3D volume rendered images shows the spinal dysplasia (arrows) and accompanying scoliosis. These images can be rotated and viewed form 360 degrees.

Dural ectasia, a characteristic finding of NF-1, is an expansion of the thecal sac, which may result in posterior vertebral body scalloping and lateral thoracic meningocele formation. Widening of the spinal canal and enlargement of the neural foramen is often associated with dural ectasia [7,8,23,24]. Destabilization of the vertebrae may also occur, leading to spontaneous subluxation or dislocation [25,26]. Spinal meningoceles, which are observed in 60-85% of patients with NF-1 can cause headache, coughing, dyspnea, sometimes with back pain with or without motor and sensory symptoms and should be treated surgically when possible [27-29]. MDCT and VR were particularly useful in evaluating these bony abnormalities. This included bony dysplasia in all cases with plain radiographs and MRI, and in 2 of 4 cases when VR was compared to MDCT (Figure 1). This related to better definition of the bony structures with MDCT, thinner section imaging in the coronal and sagittal planes, and the VR images that allowed a compete view of the spine on 1 image, and rotation of the images allowing visualization from 360 degrees.

Spinal deformities associated with NF-1 may be dystrophic or nondystrophic [8,9,30,31]. Although non-dystrophic deformities resemble idiopathic scoliosis, progression to the dystrophic form can occur, and careful follow up is necessary [9,32]. Features of dystrophic deformities include vertebral scalloping, rib penciling and spindling of the transverse process, wedging of one or more vertebral bodies, paraspinal or intraspinal soft tissue masses, subluxed or displaced vertebral bodies, frame enlargement and defective pedicles [3,5]. Dystrophic spinal deformities may exhibit severe angulations and rapid progression [7,9,31,33,34]. As a general rule, the more severe the dystrophic changes identified in the vertebral bodies, the higher the likelihood that the scoliotic curvature will deteriorate. Surgical management of dystrophic spinal neurofibromatosis deformities requires an early and aggressive approach [17,34-37] and is a demanding procedure [5,35,38,39], so precise delineation of spinal abnormalities is absolutely essential (Figure 1). The variable and sometimes rapid progressive course of dystrophic scoliosis in children with NF-1 complicates management [8]. In our study, the MDCT study was helpful in all cases for the evaluation of bony dysplasia and fusion, especially in patients with scoliosis.

The separation of NF-1 associated scoliosis into dystrophic and nondystrophic curves is made with plain radiographs and may suffer from the inability of such imaging to identify early bone changes [9]. If dystrophic changes are noted on plain radiographs, MRI has been considered essential to further investigate the intraspinal contents, particularly when surgical management of scoliosis is anticipated [5,6]. However, the interpretation of MRI findings is difficult, because of the complex three plane deformity of kyphoscoliotic curvatures [5,35]. In our series, MDCT with VR was helpful in 5 of 7 patients with an abnormal spinal curvature compared to plain radiographs, and in 10 of 11 cases when compared to MRI. In addition, the VR images were graded as helpful in 24 of 31 patients with an abnormal spinal curvature when compared to the MDCT study alone. We found the VR images to be most helpful in the evaluation of a complex spinal curvature, as the entire spine is visualized on a single image that can be rotated to allow visualization from 360 degrees (Figure 1C, D). The VR images are less helpful if the abnormal curvature is not complex and does not extend out of the sagittal or coronal plane.

**Figure 2:**
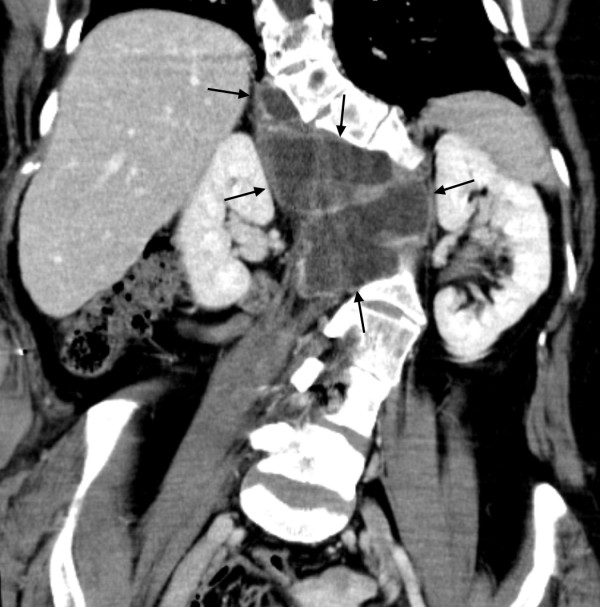
**A 19 year-old female (Patient #1) with enlarging left plexiform neurofibroma which transformed to a malignant peripheral nerve sheath tumor. *A***. Plain radiograph shows a large left neck plexiform neurofibroma (large arrows) and suggests destruction of the posterior elements at C7 (small arrows). ***B***. Coronal T1 without contrast shows the large left plexiform neurofibroma (arrows) with poor delineation of the bony elements. ***C***. Axial MDCT without contrast demonstrates destruction of the T1 vertebral body, lamina and transverse process as well as the T1 rib (arrows). ***D***. 3D volume rendered images with destruction of the bony structures at the C7 and T1 vertebral bodies (black arrows) and adjacent left T1 rib (white arrows).

Although the risk of malignant transformation is low, estimated to be about 2-6%, [40,41] this has been described as a major element in the natural history of NF-1 [42]. It is believed that most malignant peripheral nerve sheath tumors arise from plexiform neurofibromas and the most common age of presentation is 20–50 years old [5,41,43]. The goal is to achieve complete removal of the tumor whenever it is safe to do so, because the recurrence rate is high in partially resected tumors [23]. MDCT and the VR images may be performed in patients with both benign and malignant nerve sheath tumors to assess for bone destruction, which, while not diagnostic, is suggestive of a malignant lesion [44] (Figure 2). In our study, we found the MDCT studies to be helpful in the evaluation of bone remodeling and destruction related to nerve sheath tumors. For assessment of the nerve sheath tumor, the MDCT study demonstrated nerve sheath tumors better than plain radiographs, however, MDCT was only of benefit when compared to MRI for large lesions that were outside of the field of view on MRI.

**Figure 3:**
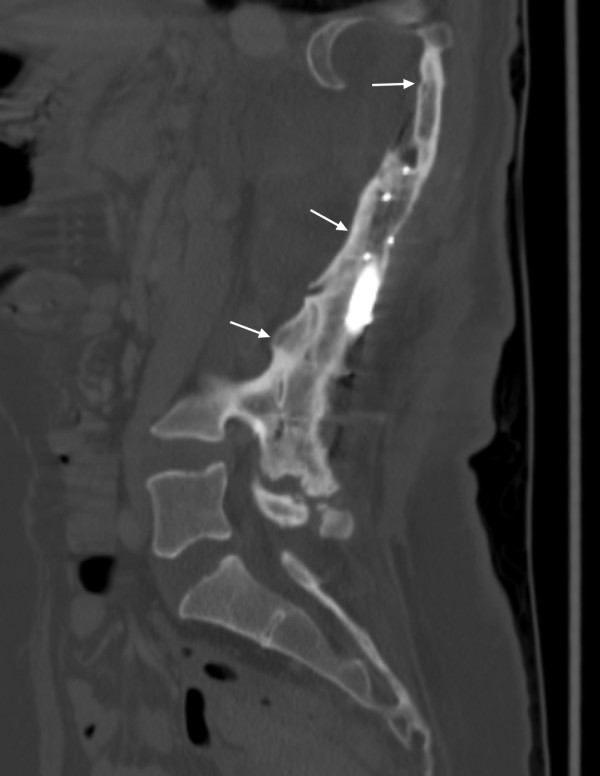
**36 year-old female (Patient #16) with levoscoliosis, status post surgical correction. *A***. Sagittal FSE T2 without fat saturation demonstrates significant susceptibility artifact from surgical instrumentation. ***B***. Coronal MDCT with contrast (soft tissue window) demonstrating the scoliotic curvature and lateral meningoceles at multiple levels (arrows). ***C***. Sagittal MDCT with contrast (bone window) shows the bony arthrodesis from surgical fusion (arrow). ***D***. 3D MDCT with translucent display demonstrating the position and integrity of the surgical construct and the bony arthrodesis (arrows).

Postoperatively, because of a high incidence of failure of fusion, some authors recommend routine tomographic evaluations 6 months after spinal surgery and a direct augmentation procedure if evidence of failure is present [28,39]. Our results suggest that MDCT imaging, especially utilizing the VR images with translucent display (Figure 3), is well suited for the evaluation of the post-operative spine in patients with NF-1. This is because the surgical instrumentation and adjacent bony structures are better visualized due to a significant reduction in streak artifact related to metallic hardware [45,46], a particular advantage over MR imaging where there is decreased visualization of the metallic construct and the osseous structures (Figure 3). This is maybe helpful in the evaluation of instrumentation failure, including loosening and breakage. In 24 of 31 patients with surgical instrumentation, the VR images with translucent display aided the MDCT in evaluation of the surgical construct. The VR images were helpful if the instrumentation extended over multiple vertebral body levels, but less helpful for shorter segment instrumentation, such as 2-level anterior cervical fusion.

The patients in our series represent a small portion of all patients with NF-1 seen at our institution. MDCT imaging is not performed in all patients, mostly in patients with more severe forms of scoliosis and spinal dysplasia and when clinical symptoms require further imaging. Therefore, our results may have a selection bias and may appear to exaggerate the benefit of MDCT in this patient population. However, we have demonstrated areas where MDCT is beneficial in the evaluation of abnormalities related to NF-1, including bony abnormalities such as dural ectasia, fusion, and dysplasia, abnormal spinal curvature, and spinal instrumentation.

The MDCT study has higher costs than plain radiographs; however, we have demonstrated the benefit of MDCT and VR images over plain radiographs for patients with NF-1 in the evaluation of bony abnormalities, scoliosis, and the assessment of surgical instrumentation. Further studies, including cost-benefit analysis, may be undertaken to help determine the exact role of this technique for this patient population.

There may be an increased risk of tumor transformation due to radiation from the MDCT studies. However, we feel that the risks are outweighed by the benefits to patients with NF-1, as MDCT provides additional information in the evaluation of the spine in assessment of the aforementioned abnormalities. Failure to obtain a complete evaluation of the spine in patients with complex abnormalities may prove detrimental in surgical planning and following spinal instrumentation. As previously stated, the VR images, which provide additional information over the MDCT study alone, are generated by computer manipulation of the axial raw without the need for additional radiation to the patient. Efforts are currently undergoing to further reduce the MDCT radiation dose [47,48].

Herein, we have sought to illustrate the use of MDCT imaging of the spine in patients with NF-1 and demonstrate the role of MDCT to MR imaging in this setting and its role as a complementary examination. Further studies may be undertaken with larger number of the assessed abnormalities, to determine the exact role of MDCT and alternate imaging modalities in patients with NF-1, and this study may serve as the basis for those studies. In addition, evaluation of MDCT imaging may provide an opportunity for further research to refine understanding of scoliosis in patients with NF-1.

## Conclusion

MDCT with VR is beneficial for the evaluation of the spine in patients with NF-1. Bony abnormalities, abnormal spinal curvature, and surgical instrumentation are particularly well demonstrated with the VR images. These abnormalities may be severe and associated with significant consequences if not appropriately addressed. Accurate imaging is essential in the evaluation of these patients with complex anatomy for diagnostic purposes and pre-operative and post-operative assessment.

## Abbreviations

NF-1: Neurofibromatosis type 1; CT: Computed tomography; MDCT: Multidetector CT; 2D: Two-dimensional; 3D: Three-dimensional; VR: Volume rendered.

## Competing interests

The authors declare that they have no competing interests.

## Authors’ contributions

JMD conceived of the study, participated in its design and coordination, and helped to draft the manuscript. YMMM participated in its design and coordination, and helped to draft the manuscript. LK conceived of the study, participated in its design and coordination, and helped to draft the manuscript. JMS participated in its design and coordination, and helped to draft the manuscript. IEM participated in its design and coordination, and helped to draft the manuscript. NGT conceived of the study, participated in its design and coordination, and helped to draft the manuscript. All authors read and approved the final manuscript.
